# Entrainment of superoxide rhythm by menadione in HCT116 colon cancer cells

**DOI:** 10.1038/s41598-019-40017-7

**Published:** 2019-03-04

**Authors:** Uma Kizhuveetil, Meghana V. Palukuri, Priyanshu Sharma, Devarajan Karunagaran, Raghunathan Rengaswamy, G. K. Suraishkumar

**Affiliations:** 1 Department of Biotechnology, Bhupat and Jyoti Mehta School of Biosciences building, Indian Institute of Technology Madras, Chennai, 600036 India; 2 Department of Chemical Engineering, Indian Institute of Technology Madras, Chennai, 600036 India

## Abstract

Reactive oxygen species (ROS) are primary effectors of cytotoxicity induced by many anti-cancer drugs. Rhythms in the pseudo-steady-state (PSS) levels of particular intracellular ROS in cancer cells and their relevance to drug effectiveness are unknown thus far. We report that the PSS levels of intracellular superoxide (SOX), an important ROS, exhibit an inherent rhythm in HCT116 colon cancer cells, which is entrained (reset) by the SOX inducer, menadione (MD). This reset was dependent on the expression of p53, and it doubled the sensitivity of the cells to MD. The period of oscillation was found to have a linear correlation with MD concentration, given by the equation, T, in h = 23.52 − 1.05 [MD concentration in µM]. Further, we developed a mathematical model to better understand the molecular mechanisms involved in rhythm reset. Biologically meaningful parameters were obtained through parameter estimation techniques; the model can predict experimental profiles of SOX, establish qualitative relations between interacting species in the system and serves as an important tool to understand the profiles of various species. The model was also able to successfully predict the rhythm reset in MD treated hepatoma cell line, HepG2.

## Introduction

Cancer treatment or management strategies such as chemotherapy and radiotherapy are predominantly based on the cytotoxicity caused by intracellular reactive oxygen species (ROS) such as superoxide (SOX) and hydroxyl radicals^1–4^. The efficacy of such strategies can be significantly improved, which would also reduce the undesirable side effects^5,6^. Although the effectiveness of anticancer therapeutics is known to be dependent on the circadian timing of their administration^7–9^, the chronotherapeutic methods of drug administration are not widely used probably due to a limited understanding of the underlying rhythms.

Biological rhythms have long been known to have effects on drug metabolism^10,11^. More than a hundred chemicals including anticancer drugs have been reported to have circadian changes in their pharmacokinetics^12,13^. Variation in drug pharmacokinetics, the circadian control of drug metabolism and cellular detoxification have been reported to be responsible for the temporal variation in toxicity and efficacy of drugs^14^.

However, the rhythmic variations of ROS, the primary effectors of cytotoxicity, and their effect on cancer drug effectiveness have not been reported thus far, probably due to the high reaction rates associated with ROS. However, it is known that the pseudo-steady-state (PSS) levels of ROS provide valuable information, which can be used to manipulate slower processes with much larger characteristic times; e.g. the resets in the rhythms of PSS levels of ROS significantly improved lipid productivity in microalgae^15^.

In this work, we report for the first time, a circadian rhythm in intracellular specific SOX levels in the colon cancer cell line, HCT116. No reports are available to date on rhythms of SOX in cancer cells. Further, we have studied the reset of the inherent SOX rhythm to a higher frequency by menadione (MD), which needs the expression of p53 in the cell.

MD is a well-established intracellular SOX generator^16,17^, which generates SOX by a redox cycling mechanism involving an enzyme catalysed single electron reduction of MD to its corresponding semiquinone (SQ) followed by the spontaneous cycling back to MD. This mechanism of redox cycling has been reported to be common to many well-known anticancer drugs^18–20^.

We have also developed a mathematical model to simulate the p53 dependent reset of SOX rhythm observed in HCT116 wt. The model generated results were found to be consistent with our experimental observations, which strengthen the possibility that the rhythms of SOX play a considerable role in the mode of action of MD in HCT116 wt.

## Development of a mathematical model for the reset of SOX rhythm In HCT116 colon cancer cells

We have developed a mathematical model to better understand the mechanism of the reset in SOX rhythm by MD in HCT116 wt cells. MD is known to affect the extracellular signal -regulated kinase (ERK)^21,22^ and the protein p53^23^, which have tumour relevance. Therefore, an ERK–p53 pathway was chosen for the model development. The molecular interactions suggested by Li *et al*.^24^ through their experimental results for the effect of selenite, a redox active drug, in the apoptosis of NB4 cells, were modified by the addition of a feed-back loop for the regulation of SOX by MnSOD to derive our model for the p53–dependent, MD induced reset of SOX rhythm.

The proposed mathematical model can be said to capture the major contributing factors of the modelled phenomena if the model results are close to the experimental observations. However, the experimental system, due to its high level of complexity, may have aspects which are unaccounted for in the model. It is known that a successful mathematical model is only an abstraction of the most important features of the system, not all^25^. The assumptions made during the derivation of the current model are given below.

### Assumptions

It was assumed that oxygen levels are saturated at a value of 20 μM^26^ as the system being considered was an *in vitro* cell culture. The translocation rate of ERK from the cytoplasm to the nucleus was considered to be much higher than the translocation rate in the reverse direction. The translocation rate of ERK into the nucleus was assumed to be second order, dependent on both ERK and SOX concentrations, instead of a first order reaction^27^ that does not consider SOX mediated activation of ERK. These assumptions lead to the use of a few conservation relations in the model (see Supplementary Fig. S1). The transcription of *sod*2 gene was assumed to be a single activator mediated activation of transcription by p53-P following Hill kinetics^28^. A Hill coefficient of 2 accounted for the positive cooperativity of binding of p53 to the DNA^29^. With these assumptions in place, the following reactions were considered for the model.

### Reactions

MD acts via the production of SOX anion radicals in the cells through a redox cycling mechanism^16,17^ comprising of two main steps. The first step involves the conversion of MD to its corresponding semiquinone (SQ) catalysed by the enzyme cytochrome P450 reductase (CP450R), which follows bi-substrate enzyme kinetics^30^ with NADPH as the second substrate. This is followed by the second step that involves the spontaneous reaction of SQ with oxygen to generate SOX^31^. The SOX thus generated affects the activation of ERK^32^ and causes its translocation into the nucleus, with the reaction kinetics dependent on both ERK and SOX concentrations. The translocated ERK acts as the catalyst for the activation of the transcription factor p53 by phosphorylation^33,34^ to form p53-P. Alongside, the de-phosphorylation of p53-P by nuclear p53 phosphatases also occur. Both phosphorylation and de-phosphorylation reactions follow Michaelis-Menten (MM) kinetics^35–37^. The activated p53-P binds to the promoter and acts as a transcription factor for the gene *sod*2 which codes for the protein Mn SOD^38–40^. Mn-SOD, formed from the m*sod*2 mRNA, is an enzyme which follows MM Kinetics^41^ and catalyses the dismutation of SOX to hydrogen peroxide and oxygen and thus reduces the cellular SOX levels. The NADP^+^ formed during the conversion of MD to SQ is cycled back to NADPH by cellular NADP^+^ reductases such as Ferredoxin NADP reductase (FNR)^42^.

The equations were stoichiometrically balanced by considering degradations of mRNA and protein back to the cellular metabolite pool. Ordinary differential equations (ODEs) were derived for each of the reacting components, according to the general formula

1 $$\frac{d\,[X]}{dt\,}=rate\,of\,generation\,of\,X-rate\,of\,consumption\,of\,X\,$$

The various parameters used for the model are as follows: the amplitude of the decaying sine wave (A), concentration of chromatin DNA (D_n_), the constants for menadione (K_M_), NADPH (K_N_), SOX (K_SOX_), p53 (K_p53_), p53-P (K_p53-P_) and NADP^+^ (K_NADP_^+^), all with units of µM, the maximal possible transcription initiation rate (I_max_, h^−1^), the rate constant for conversion of SQ to SOX (k_1_, µM^−1^ h^−1^), the rate constant for conversion of SOX to SQ (k_m1,_ µM^−1^ h^−1^), the rate constant for the generation of p53 (k_p_, h^−1)^, the turnover number for Mn-SOD (k_2_, h^−1^), the turnover number for the phosphorylation reaction of p53 (k_3_, h^−1^), the rate constant for nuclear translocation of ERK (k_t1_, µM^−1^ h^−1^), the translation rate constant (k_tr_, h^−1^), the mRNA turnover rate constant (k_dm_, h^−1^), the protein turnover rate constant (k_dP_, h^−1^), the damping factor for the decaying sine wave (L, h^−1^), the dimensionless Hill coefficient for binding of p53-P to DNA (n_a_), the ratio of nuclear volume to cytoplasmic volume of a cell (n_v_), phase of the decaying sine wave (phi, radians), the initial velocity for CP450R, with menadione and NADPH (v_1,_ µM h^−1^) and the maximal rates for conversion of MD to SQ (V_m1_), p53-P to p53 (V_m4_) and NADP^+^ to NADPH (V_m5_), all in µM h^−1^.

The reactions, rate equations and mass balances used in the model are given in Tables 1 and 2.

**Table 1:** Reactions and rate equations used for the model.

Reaction	Rate equation
$MD+NADPH\mathop{\to }\limits^{CP450R}SQ+NAD{P}^{+}$	${r}_{1}=\frac{{V}_{m1}[MD][NADPH]}{{K}_{N}[MD]+\,{K}_{M}\,[NADPH]+[MD]\,[NADPH]}$
$SQ+{O}_{2}\underset{{k}_{m1}}{\overset{{k}_{1}}{\rightleftharpoons }}MD+SOX+{H}^{+}$	${r}_{2}={k}_{1}[{O}_{2}][SQ]-{k}_{m1}[MD][SOX]$
$ER{K}_{c}+SOX\mathop{\to }\limits^{{k}_{t1}}+\,ER{K}_{n}+\varnothing$	${r}_{3}={k}_{t1}{[ERK]}_{C}[SOX]$
$P+p53\underset{phosphatase}{\overset{ERK2}{\rightleftharpoons }}\,p53-P$	${r}_{4}=\frac{{k}_{3}{n}_{v}n{[ERK]}_{c}[p53]}{{K}_{p53}}-\frac{{V}_{4}[p53-P]}{{K}_{p53-P}+[p53-P]}$
$2SOX+2{H}^{+}\mathop{\to }\limits^{Mn-SOD}+\,{H}_{2}{O}_{2}+{O}_{2}$	${r}_{5}=\frac{{k}_{2}[Mn-SOD][SOX]}{{K}_{SOX}+[SOX]}$
$\varnothing +p53-P+DNA\to mSOD2$	${r}_{6}=\frac{{I}_{max}[{D}_{n}]{[p53-P]}^{{n}_{a}}}{{K}_{p53-P}^{{n}_{a}}+{[p53-P]}^{{n}_{a}}}$
$mSOD2\to \varnothing$	${r}_{7}=\,{K}_{dm}[mSOD2]$
$\varnothing +mSOD2\,\to Mn-SOD+\,mSOD2$	${r}_{8}=\,{K}_{tr}[mSOD2]$
$Mn-SOD\,\to \,\varnothing$	${r}_{9}={K}_{dp}[Mn-SOD]$
${H}^{+}+NAD{P}^{+}\mathop{\to }\limits^{FNR}NADPH$	${r}_{10}=\frac{{V}_{5}[NAD{P}^{+}]}{{K}_{NAD{P}^{+}}+[NAD{P}^{+}]}$
$\varnothing \mathop{\to }\limits^{MD}p53$	$r11={k}_{p}[MD]$
	$${r}_{oscil}=-\,{\rm{LA}}{e}^{-Lt}+{\rm{A}}{e}^{-Lt}({\rm{wcos}}({\rm{wt}}+{\rm{phi}})-{\rm{Lsin}}({\rm{wt}}+{\rm{phi}}))$$

**Table 2:** Mass balance equations used in the model.

$$\frac{d\,[MD]}{dt}=-\,{r}_{1}+{r}_{2}-{r}_{11}$$
$$\frac{d\,[NADH]}{dt}=-\,{r}_{1}+{r}_{10}$$
$$\frac{d\,[NAD{P}^{+}]}{dt}={r}_{1}-{r}_{10}$$
$$\frac{d\,[SQ]}{dt\,}={r}_{1}-{r}_{2}$$
$$\frac{d\,[SOX]}{dt}={r}_{2}-{r}_{3}-2{r}_{5}+{r}_{oscil}$$
$$\frac{d\,{[ERK]}_{c}}{dt}=-\,{r}_{3}$$
$$\frac{d\,{[ERK]}_{n}}{dt}={n}_{v}{r}_{3}$$
$$\frac{d\,[p53]}{dt}={r}_{11}-{r}_{4}$$
$$\frac{d\,[p53-P]}{dt}={r}_{4}$$
$$\frac{d\,[mSOD2]}{dt}={r}_{6}-{r}_{7}$$
$$\frac{d\,[Mn-SOD]}{dt}={r}_{8}-{r}_{9}$$

The formation of SOX in the cell is a complex process^43^. To account for the endogenous levels of SOX in the cell, a decaying sine wave term was introduced in the mass balance for SOX. Interestingly, the experimental data showed that the time period has a linear correlation with the MD concentration only when p53-P is present. Incorporating this relation, we propose the following equation for the frequency:

2 $$\omega =\frac{2\pi }{a-b[MD]p}$$

The parameters, a and b are estimated for the model using linear regression of initial MD concentration vs average time period (obtained from the PAST analysis), for the data corresponding to the wt case as shown in Supplementary Fig. S2. Although obtained from the dataset corresponding to the wt case, interestingly, the parameter ‘a’ is found to predict the experimental observation in the case corresponding to the absence of p53 as well.

The initial concentration used for each component is given in Supplementary Table S1.

Optimized parameter values were obtained through the parameter estimation techniques defined in detail in the supporting information (see Supplementary Table S2). It was ensured that the values are biologically meaningful – they were obtained from a search space containing the biological ranges possible for each parameter. In addition to being biologically relevant, the parameters need to account for experimental errors. So, the phase, which can account for minor experimental errors in synchronization, was adjusted for each case to ensure a better fit. The phase values for the various cases are reported in Supplementary Table S3.

## Results

### Endogenous SOX levels in HCT116 cells possess a circadian rhythm independent of p53 status

The SOX levels in the untreated HCT116 cells were determined as described in the materials and methods section, through dihydroethidium (DHE) fluorescence with improved strategies to reduce interferences. The software, PAST^44^, was used to analyse the data using a Lomb-Scargle periodogram^45^ (LSP) analysis to determine rhythms in the temporal variation of SOX in the wt and p53−/− variants of HCT116. Both the cell variants showed presence of an inherent circadian rhythm for SOX (Table 3, Supplementary Figs S3a, S4a). The peak frequencies obtained were 0.042 and 0.044 h^−1^, respectively, for wt and p53−/− cells. The period of oscillation, calculated as the inverse of the peak frequency, was about 24 h for wt cells and about 22 h for p53−/− cells.

**Table 3:** Frequency comparison of SOX time profiles.

Case	HCT116 wt		HCT116 p53−/−	
	Experimental (h^−1^)	Model (h^−1^)	Experimental (h^−1^)	Model (h^−1^)
Control	0.042	0.046	0.044	0.044
MD = 3 μM	0.047	0.052	0.044	0.044
MD = 6 μM	0.068	0.060	—	0.044
MD = 9 μM	0.065	0.071	0.047	0.044
MD = 10 μM	—	0.079	—	—
MD = 12 μM	0.107	0.090	0.047	0.044
MD = 15 μM	0.112	0.122	0.044	0.044
MD = 30 μM	—	—	0.047	0.044

The experimental values are given for p < 0.05 using the software PAST. For the individual p values, see Supplementary Figs S3 and S4.

### MD induces a p53-dependent reset in SOX rhythm in HCT116 cells

**Figure 1:**
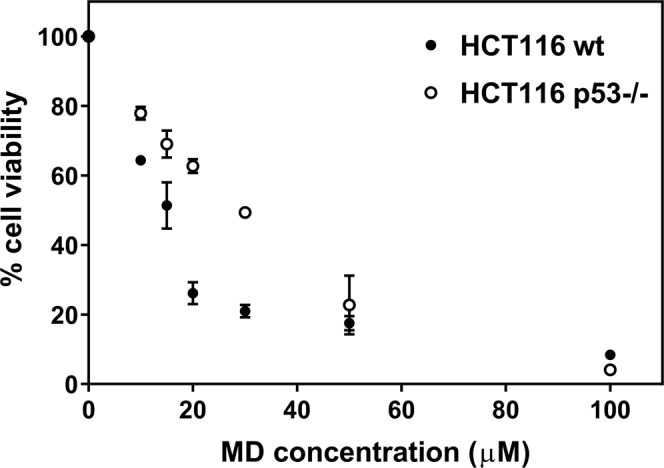
Variation of cell viability of HCT116 wt and p53−/− cells with increasing concentrations of menadione (MD). The cells were seeded at 1 × 10^4^ cells per well. The cell viability was measured using MTT, 48 h post drug treatment. The values are expressed as mean ± SD, n = 3. Paired two tailed Student’s t test showed the data to be significant with p = 0.049.

Both the wt and p53−/− cell variants were treated with various concentrations of MD. The IC_50_ values were determined 48 h post drug treatment (Fig. 1) using the 3-(4, 5-dimethylthiazol-2-yl)-2, 5-diphenyl tetrazolium bromide (MTT) assay, and the IC_50_ values were in the earlier reported range^46^. The IC_50_ value was around 15 µM for HCT116 wt cells whereas the efficacy of the drug on the p53−/− variant was 50% less with an IC_50_ value of around 30 µM.

**Figure 2:**
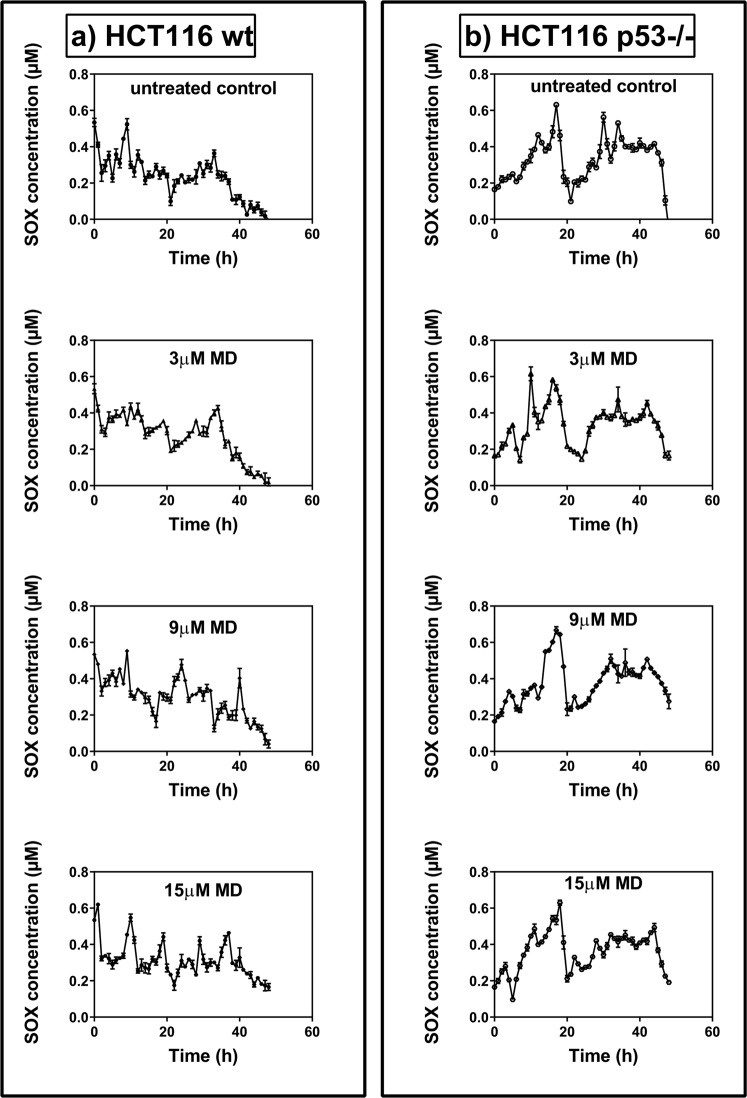
Temporal variations in SOX levels in (**a**) HCT 116 wt cells and (**b**) HCT 116 p53−/− cells for untreated control, 3 µM, 9 µM and 15 µM menadione (MD) treatment. The cells were seeded in 6 well plates and synchronized for 24 h in serum free medium. 0 h corresponds to the time of medium change to DMEM with 10% FBS with (treated) or without (untreated) MD. The time where SOX levels peak are different for different concentrations of MD in the wt cells whereas the trend is unchanged in the p53−/− cells. The values are expressed as mean ± SD, n = 3. The data was shown to be significant using two way ANOVA with p < 0.001.

The temporal variations of SOX levels were markedly different for wt and p53−/− cells (Fig. 2). The LSP analysis using PAST for wt cells showed that the frequency of SOX rhythm shifted from 0.042 h^−1^ for untreated cells to 0.112 h^−1^ for 15 µM MD treated cells. However, no significant change in rhythm was observed for the p53−/− cells (see Table 3, Supplementary Figs S3 and S4).

### The mathematical model predicts SOX rhythm reset

**Figure 3:**
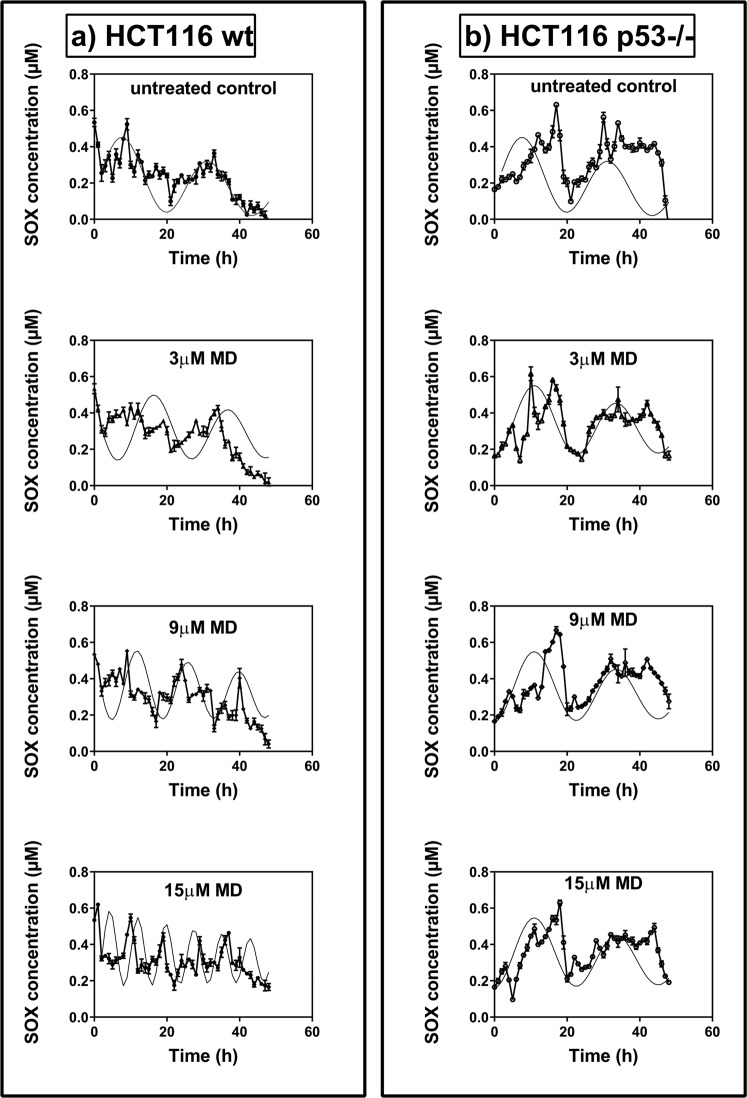
Comparison of experimental and model derived profiles of superoxide (SOX) in HCT116 wt cells and p53−/− cells. The experimental values are expressed as mean ± SD, n = 3. The experimental data was shown to be significant using two way ANOVA with p < 0.001.

The model generated profiles were found to fit the experimental data well for all the initial MD concentrations, for both wt and p53−/− cases (Fig. 3). The peak frequencies for the experimental and model generated time series were obtained using spectral analysis with LSP and are reported in Table 3.

The model was also used to make new predictions for a different initial MD concentration with and without p53 (Supplementary Fig. S5). The frequencies of these cases are also reported in Table 3. We find that they fit into the trends followed by the frequency as a function of MD induced cytotoxicity.

#### Robustness and sensitivity analysis

The robustness limits for all the parameters, along with the species affected the most by each parameter are reported in Supplementary Table S4. The initial MD concentration of 15 μM in the presence of p53 was used for the analysis. The robustness bound used was 20% as it represented a good span of the experimental data, as indicated in Supplementary Fig. S6.

### Experimental investigations into aspects of the mathematical model

**Figure 4:**
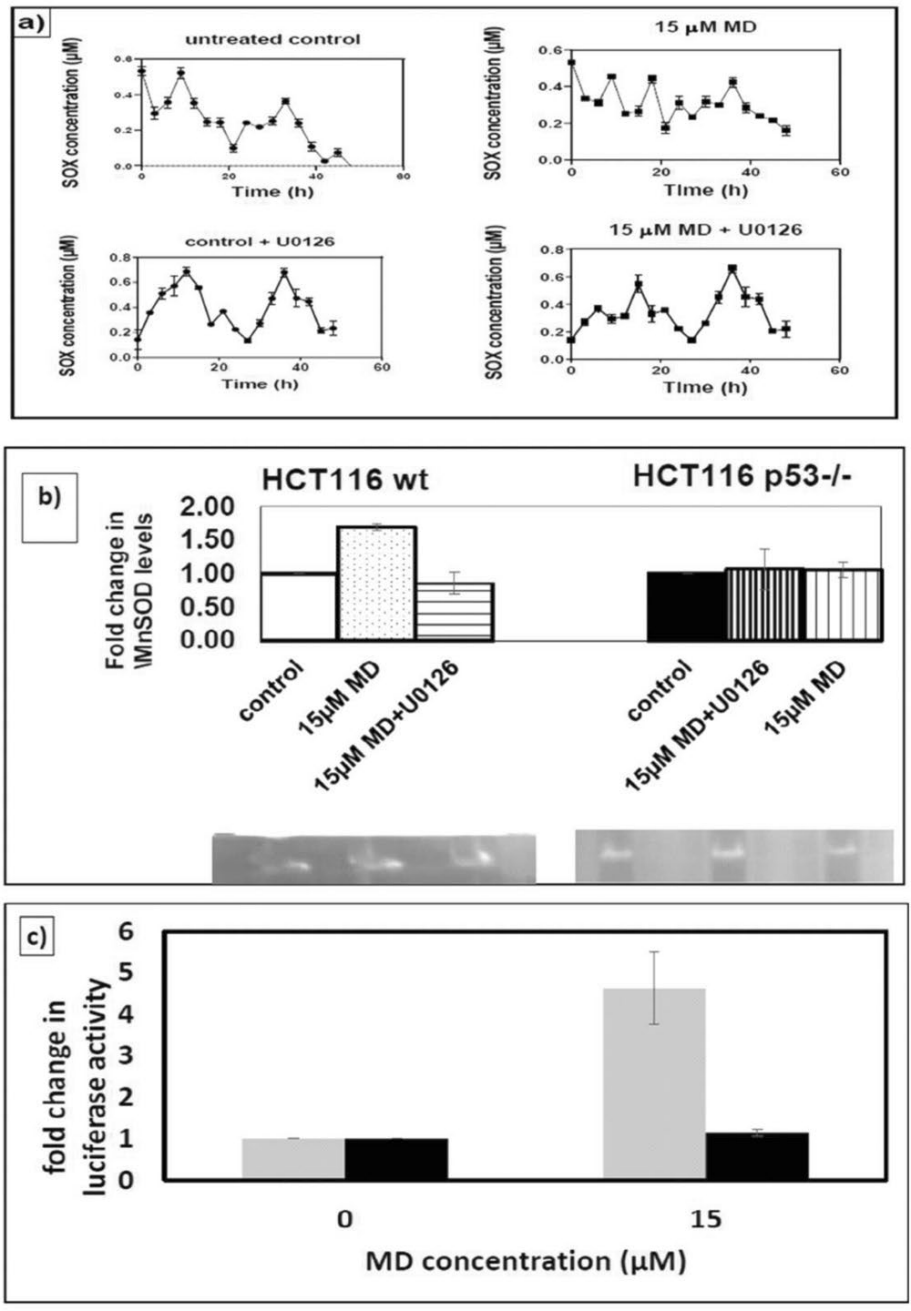
(**a**) Near circadian rhythmicity of SOX levels in HCT116 wt cells pre-treated with U0126 to inhibit ERK activation. The control rhythm is not affected, whereas the menadione induced rhythm reset does not occur when ERK activation is inhibited (**b**) In gel activity assay for manganese superoxide dismutase (MnSOD) in HCT116 wt and p53−/−. The graph shows the fold change in the MnSOD levels in menadione treated cells, pre-treated or untreated with U0126 for two hours to inhibit ERK activation, as compared to its respective untreated control for wt and p53−/− cells. The analysis was done using ImageJ software. The fold changes shown are the band intensities which have been normalized to their respective control values. The full length images are given in Supplementary Fig. S12. (**c**) Fold changes in p53 luciferase activity in HCT116 wt cells at the indicated concentrations of menadione, with (black) or without (grey) U0126 pre-treatment to inhibit ERK activation. The values have been normalized to their respective control values to obtain the fold changes. Two-way ANOVA was used to analyse the significance (p < 0.001) in the differences. The experimental values are expressed as mean ± SD, n = 3.

When the HC116 wt cells were pre-treated for 2 h with 10 µM of U0126 (inhibitor of mitogen-activated protein kinase kinase (MEK), the kinase which activates ERK) in a serum starved medium, there was a drastic effect on the rhythm reset observed in MD treated cells with the period of oscillation shifting from 8.9 h to 21.8 h for 15 µM MD treated cells. (Fig. 4a).

To determine whether Mn-SOD levels showed any up-regulation on MD treatment, in gel activity analysis was performed with the cell lysate (Fig. 4b). The results were similar to model predicted results, with Mn SOD levels being up-regulated by about 1.6 fold in the HCT116 wt cells treated with MD, 48 h after MD treatment and the p53−/− cells showing no significant change upon MD treatment. Pre-treatment of the cells with U0126, resulted in decrease in MnSOD levels in 15 µM MD treated cells to a level similar to that of control in wt cells, whereas they were unaffected in p53−/− cells.

MD treated cells showed a 4 fold increase in the activated p53 levels as indicated by pG13 luciferase reporter assay, which significantly decreased to 1.1 fold upon pre-treatment with U0126 (Fig. 4c). Further, the western blots of p53, ERK and pERK were analysed (Supplementary Fig. S7). The results show that the p53 levels between control and MD treated HCT116 wt cells were not significantly different at least up to 24 h, whereas MD significantly up-regulated ERK and pERK levels by 1.4-fold and 1.3-fold, respectively. But, p53 levels were down-regulated upon treatment with U0126 (Supplementary Fig. S7).

Since the model predicted variations were close to the actual observed changes in case of experimentally determined concentrations, the variations in the other species involved could also be reliable. Therefore, the model serves as an important tool for elucidating the reaction mechanism of MD by predicting the time profiles of the various species involved in this action, which would be difficult to determine experimentally. These profiles are given in Supplementary Fig. S8.

We have observed that the redox cycling compound, doxorubicin, also induces SOX rhythm reset in HCT116 wt cells, which is reversed upon inhibition of ERK activation (Supplementary Fig. S9). SOX rhythms in the hepatoma cell line HepG2 which expresses wt p53 were also analysed. HepG2 shows an inherent SOX rhythm of 25.6 h which was reset to 11 h upon treatment with MD (Supplementary Fig. S10).

## Discussion

In this work, we report for the first time, a circadian rhythm in intracellular specific superoxide levels in cancer cells. The model cell line used for most of the studies was a human colon cancer cell line, HCT116. The temporal variations of SOX levels in the colon cancer cell line HCT116 which expressed p53 (wt) as well as its p53−/− variant, showed near-circadian rhythms of 24 h and 22 h, respectively. The ROS levels were higher in cancer cells compared to normal cells. However, survival of cancer cells requires regulation of these ROS levels below a certain threshold^47,48^. Since SOX have been reported to be important signalling molecules in the cell, the presence of these endogenous circadian rhythms suggests a possibility for tight regulation in the redox homeostasis to enable the cancer cell survival. Since ROS are crucial regulators of cell death^1–4^, the above leads to a speculation that alterations in SOX rhythms could be a mechanism for inducing cancer cell death. Also, ROS have been implicated in the cell death induced by multiple anti-cancer agents^1–6^. Thus, a better understanding could lead to the use of ROS rhythms as a generic target for better effective cancer chronotherapy.

MD is a well-established SOX generator^16,17^ that generates SOX through a redox cycling mechanism. The generation of additional SOX in the system by MD perturbs the SOX homeostasis in the cells and could be used to study the variations of SOX rhythm. We found that the exposure of HCT116 cells to a single pulse of MD reset the SOX rhythm to a higher frequency; the frequency increased with increase in MD concentration. Also, we found that the reset was dependent on the presence of the tumour suppressor protein, p53, since the p53−/− cell variant did not show any reset in the SOX rhythms. It is interesting to note that the cytotoxicity induced by MD in HCT116 cells was also p53 dependent with the p53−/− variant requiring double the concentration (IC_50_ = 30 µM) of MD to induce 50% cell death as compared to HCT116 wt (IC_50_ = 15 µM).

Experimental evidence is available for the involvement of an ERK–p53- MnSOD pathway in the action of selenite^24^ treatment on cancer cells. Also, ERK has been reported to be activated by MD^21,22^ and multiple other redox cycling anti-cancer drugs^49,50^. Since we have found the presence of p53 to be required for the rhythm reset, the possible involvement of an ERK –p53-MnSOD pathway in the SOX rhythm reset was hypothesized. MnSOD also was an ideal component to be involved in a feedback regulation of SOX, implied by the oscillatory nature of SOX concentrations. Since MD has been well studied, the use of MD for the model provided good initial values for optimization of parameters.

We found that the system was extremely sensitive to changes in n_a_ (the Hill coefficient), as it altered the functional form of the system. The system was highly sensitive to parameters Im, k_3_, K_p53_, V_m4_, K_t1_, n_v_, phi, k_1_ and k_m1_, moderately sensitive to parameters A, L, k_2_, k_tr_, V_m1_, k_na_ and V_m5_, and less sensitive to the parameters K_sox_, K_p53p_, k_m_ and K_NADP_^+^. It can also be seen that the highly sensitive parameters are mostly involved in the nuclear events such as translocation of ERK or in the formation of p53-P and mSOD2.

The parameter that showed a very high sensitivity was n_a_, the Hill coefficient for the binding of p53 to the DNA. The reported literature values are between 1.7 and 2, signifying a positive cooperativity for binding of p53 to the DNA. The changes in n_a_ values, even within these limits, significantly affected the concentration profiles of multiple species, especially the transcriptional formation of *sod*2 mRNA, in the model.

The model generated as described earlier, was able to predict the reset of SOX rhythm in HCT116 wt cells. The near circadian rhythm observed in the p53−/− case for all concentrations of MD is predicted satisfactorily as well. When the HCT116 wt cells were pre-treated with U0126 to inhibit ERK activation, the observed rhythm was near circadian in both control and 15 µM MD treated cells, implying the involvement of ERK activation in the MD induced rhythm reset.

The MnSOD levels were analysed using an in gel activity assay. HCT116 wt cells showed a 1.6 fold change in the MnSOD levels upon treatment with MD, which was similar to the model predicted values. This increase in MnSOD levels were not observed in MD treated cells when the HCT116 wt cells were pre-treated with U0126. The p53−/− cell variant did not show any changes in the MnSOD levels upon treatment with MD, in the presence or absence of U0126 pre-treatment. Therefore the presence of activated ERK seems to be required for MnSOD up-regulation in HCT116 wt cells. However, the basal levels of MnSOD in p53−/− cells seem to be higher. This could possibly be due to the complex cellular regulation of SODs involving multiple signalling networks^51^. The reason for the higher basal expression of MnSOD in p53−/− variant requires further in depth investigations, which are beyond the scope of the current study.

The finding that the p53 levels were not significantly different between control and MD treated cells is consistent with the outcome of another study done by Ham *et al*.^52^ where the authors have demonstrated that p53 level remains unchanged upon MD treatment in SK-hep-1 cells. However, increased level of ERK1/2 and pERK in MD treated HCT116 cells in this study suggests the activation of ERK1/2. As ERK1/2 are well known for stabilizing and enhancing p53 transcriptional activity through phosphorylation at different sites, the increased level of luciferase activity (Fig. 4c) may suggest the increase in ERK1/2 upon MD treatment. Another study by Ham *et al*.^53^ suggests a mechanistic basis of ERK activation upon MD treatment as MD inactivates MKP-1, a phosphatase, which mediates the de-phosphorylation event of MAP and hence stabilizing the phosphorylated form. However, confirming p53 activation by MD in HCT116 cells requires detailed investigations and would be beyond the scope of the present work. Nonetheless, the present data confirm the activation of ERK by MD, thereby providing a mechanistic basis for the increased MnSOD by MD.

The model described a possible mechanism for the p53-dependent SOX rhythm reset induced by MD in HCT116 colon cancer cells. Since the model was able to explain the experimentally observed rhythm aspects, the modelled mechanism seems to be mostly responsible for the observed rhythms, although it may not be solely responsible. To check whether the model’s predictive capability could be expanded to other cancer cell lines, we studied the SOX rhythm in untreated and MD treated hepatoma cells, HepG2, which has been reported to express wt p53^54^. Interestingly, HepG2 cells also showed an inherent, near-circadian SOX rhythm of 25.6 h which was reset to about 11 h upon treatment with MD at its IC_50_ value, which were similar to the model predicted results for SOX rhythm reset in HepG2 (Fig. S10). The proposed model mechanism could also be applicable to other redox cycling chemicals such as doxorubicin which induces a SOX rhythm reset in HCT 116 wt cells, reversible upon inhibition of ERK activation (Fig. S9).

**Figure 5:**
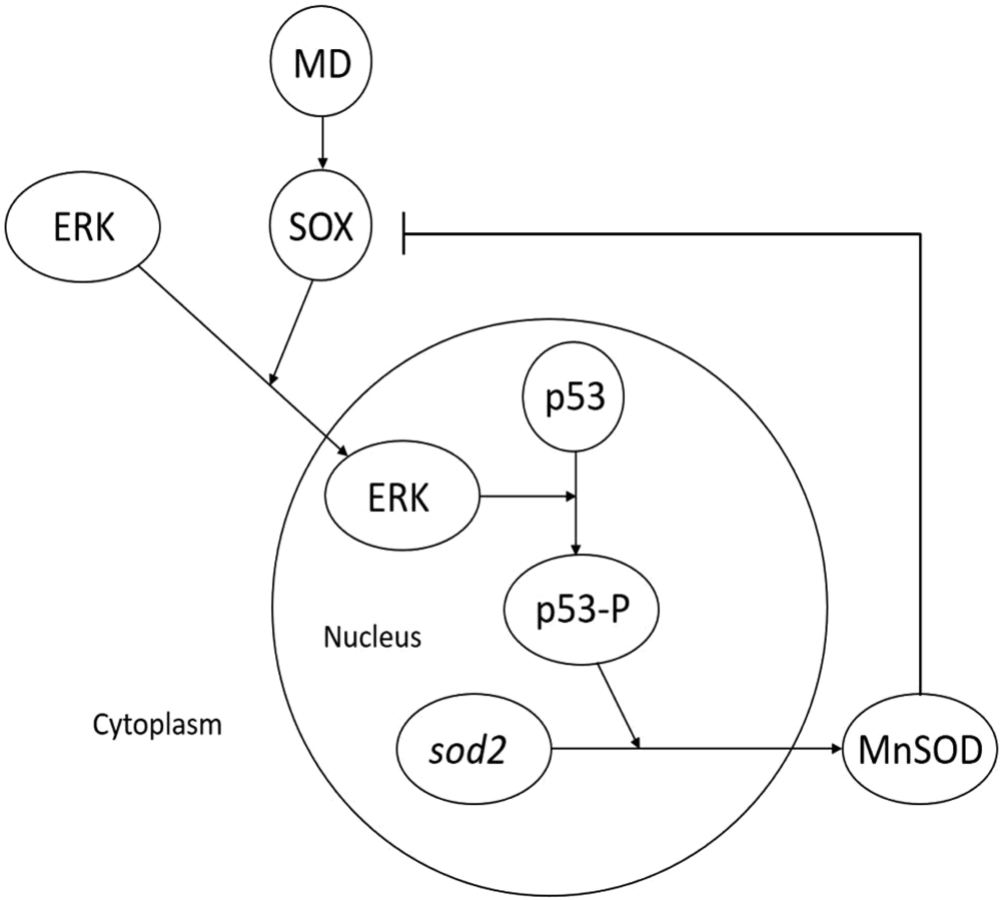
Proposed mechanism for menadione (MD) induced reset of circadian superoxide rhythms. SOX indicates superoxide, ERK – Extracellular signal-regulated kinase, p53 - Tumour suppressor protein, MnSOD - Manganese Superoxide dismutase.

In summary, the temporal variations of PSS SOX values seem to be rhythmic in HCT116 and the rhythm was reset by MD in a concentration dependent fashion in the presence of p53. Experimental SOX data from HCT116, HCT116 p53−/−, and HepG2 cell lines have been shown to be explained well by the model developed in this work. HCT116 wild type and HCT116 p53−/− cells are well known syngenic model systems, which have the same genetic background, whereas the HepG2 system is genetically different from the HCT116 colon cancer system. The model, summarized in Fig. 5, was able to predict well the results obtained from the studied syngenic systems as well as a genetically different system, which shows its general applicability. Further, in depth studies with multiple cell lines and multiple drugs need to be performed before the model could be used as a reliable tool to predict SOX rhythm reset induced by redox cycling chemicals, in general. This could also be useful in the manipulation of ROS rhythms as a target for cancer chronotherapy.

## Materials and Methods

### Growth conditions and quantification

The cell line used in this study was a human colon cancer cell line, HCT116 and the hepatoma cell line HepG2. Both the wild type (wt) and p53 null (p53−/−) variants of HCT116, were gifted by Dr. Bert Vogelstein (Johns Hopkins University, Baltimore, USA). The p53 status of the cells were validated (Supplementary Fig. S9) by western blotting^55^ using antibodies against p53 and vinculin (Cell Signaling Technology, USA). HepG2 cells were obtained from NCCS Pune, India. The cells were cultured in Dulbecco’s Modified Eagle Medium (DMEM, Himedia Labs, India) containing 10% heat-inactivated Fetal Bovine Serum (FBS, Invitrogen USA) and 1X penicillin/streptomycin solution (Himedia Labs, India). Cells were maintained at 37 °C with 5% CO_2_ in saturated humidity, and passaged upon reaching 80–90% confluency.

### Determination of MD cytotoxicity

HCT116 wt and p53−/− cells were seeded at 1 × 10^4^ cells/well in a 96-well microplate. The cells were treated with varying concentrations of MD (Sigma Aldrich, USA), 24 h after seeding. 48 h post drug treatment, the cytotoxic effect was measured using MTT (Sigma Aldrich, USA) assay^56^ by dual wavelength measurement in a microplate reader (Model 680, Bio-Rad Laboratories).

### Intracellular reactive species induction

The cells were seeded at a concentration of 4 × 10^5^ cells into 6 well dishes (Thermo Scientific, USA) in serum starved media to synchronize the cells^57^. To induce intracellular reactive species, 24 h after seeding, the spent medium was replaced with fresh DMEM containing 10% serum and varying concentrations of MD, in the test plates. Considering the drug addition time to be zero time, samples were collected every hour, up to 48 h. The control cultures, without MD were maintained at otherwise same conditions as the treated cultures.

### Intracellular SOX quantification

The intracellular SOX concentrations were quantified by a fluorescent assay using the cell permeable dye, dihydroethidium ((DHE) (Invitrogen, USA)), with an excitation/emission pair of 405/570 nm to ensure specificity^58^. The calibration curve was obtained with superoxide radicals generated by the reaction of xanthine and xanthine oxidase^59^. Both control and treated cells were trypsinized, centrifuged at 2,000 rpm for 5 min, and re-suspended in PBS, containing 10 µM of DHE, to a final concentration of 2.5 × 10^5^ cells mL^−1^. The cells were incubated at 37 °C for 30 minutes, centrifuged and re-suspended in the same volume of PBS. Fluorescence measurements were taken with 200 µL of cell suspension in each well in 96 well plates, with PBS as blank, in a multimode reader (Enspire, PerkinElmer, UK).

### Determination of superoxide dismutase levels

The two types of superoxide dismutases (SOD), Cu/Zn SOD and Mn SOD, levels were determined using an in gel activity assay^60^. Both control and treated cells were trypsinized and collected at the respective time points, centrifuged at 3,000 rpm for 5 min, washed with PBS, and re-suspended in PBS containing 0.1% Nonidet P-40 (NP-40, Himedia Labs, India) for sonication. Sonication was carried out with a sonicator (Q700, a Q-Sonica, USA) at an amplitude of 70% for a total on time of 90 seconds with pulses of 10 seconds on and 10 seconds off. 200 µg protein was loaded to each lane. The gel was stained using a mixture of Nitro Blue Tetrazolium (NBT), N, N, N′, N′-tetramethylethane-1,2-diamine (TEMED) and Riboflavin 5′ phosphate (Himedia Labs, India), the bands were developed under white light using GelDoc™ and the gel picture was analysed using ImageJ software.

### Activation of p53 through a luciferase reporter assay

A pG13 luciferase assay vector with p21 promoter construct, gifted by Dr. Ghanshyam Swarup (CCMB, Hyderabad, India), was used to determine the activation of p53 in MD treated HCT116 cells. Cells were transfected with pG13 using polyethylenimine (PEI, Polysciences Inc., USA). After transfection, the cells were pre-treated with 10 µM U0126 (Promega Life Sciences, USA) or serum free medium in case of control for 2 h and then were incubated with varying concentrations of MD. 48 h after MD treatment, the cells were collected, lysed and analysed for luciferase activity using a luminometer (Bio-Rad Laboratories, USA).

### Analysis of rhythms of SOX

The temporal data obtained for SOX were analysed using the free software PAST, which uses the Lomb Scargle Periodogram (LSP) analysis to obtain the statistically significant (p < 0.05) rhythm frequency for evenly or unevenly spaced data. The software fits the data to sinusoidal curves of increasing incremental frequencies and gives the power for each, the statistically significant frequency with the highest power being the most probable frequency for the data set.

### Mathematical modelling of superoxide rhythms

The mathematical modelling of SOX rhythms was done using MATLAB 2016b.

We developed a mathematical model comprising of 11 ordinary differential equations (ODEs) (Tables 1 and 2) and 5 species mass conservation relations (Fig. S1). The development and validation of the mathematical model involves finding a parameter set that yields SOX profiles that fit the observed experimental data. The set of ODEs comprises 23 parameters which we estimate using temporal experimental data for SOX as input. We use the case with p53 with initial MD concentration of 6 µM as the training dataset for model parameter estimation and all other datasets as test data for model validation. The model is simulated by solving the initial value problem using initial concentrations, obtained from literature (Supplementary Table S1), of the species in the system as initial conditions. Other initial conditions are used to obtain the behavior of the system under different conditions such as MD drug dosage and absence of p53.

The multi scale nature of the given system of equations makes it highly stiff, i.e. there exist nearby solutions that vary rapidly when compared to the required solution. Thus, for effective performance, we require a stiff ode solver which chooses an appropriate step size for the numerical integration of such a problem. Ode15s was used as the solver as the algorithm provides the inbuilt option of forcing only non-negative solutions, which is required in our case as we are dealing with concentrations. The genetic algorithm was found to give satisfactory parameters while maintaining good convergence and was therefore used to obtain the final parameter set. The detailed parameter estimation and robustness analysis is given in supplementary information.

### Statistical analysis

The error bars in the graphs depict standard deviation from mean for three independent experiments. The rhythm frequency was taken at p < 0.05 using the software PAST. Graph Pad PRISM 7 was used to analyse all other data by two tailed Students t test or two-way ANOVA, as applicable, to test for statistical significance of the data with alpha being set at 0.05 and p < 0.05 being considered as significant. The statistical analysis for each figure is explained in the legend.

## Supplementary information

Supplementary information

## Data Availability

The datasets generated during and/or analysed during the current study are available from the corresponding author on reasonable request.
