# Supplementary material for: Entrainment of superoxide rhythm by menadione in HCT116 colon cancer cells
Source: Sci Rep. 2019 Mar 4;9:3347. doi: 10.1038/s41598-019-40017-7 (PMC6399287; doi:10.1038/s41598-019-40017-7)
Supplement: Supplementary file 1 — Supplementary information [file 41598_2019_40017_MOESM1_ESM.pdf]

Supporting Information
for
Entrainment of superoxide rhythm by menadione in HCT116 colon cancer cells

Uma Kizhuveetil[1], Meghana V. Palukuri[2], Priyanshu Sharma[1], Devarajan Karunagaran[1], Raghunathan Rengaswamy[2] and G. K. Suraishkumar[1]*

[1]Department of Biotechnology, Indian Institute of Technology Madras, Chennai, India-600036
[2]Department of Chemical Engineering, Indian Institute of Technology Madras, Chennai, India-600036

[*]Correspondence to:
Prof. G. K. Suraishkumar
Department of Biotechnology, Bhupat and Jyoti Mehta School of Biosciences building
Indian Institute of Technology Madras, Chennai 600036 India
E-mail: gk@iitm.ac.in
Phone: +914422574105
Fax: +914422574102

Methods

Parameter estimation: Numerical integration is used to obtain the model generated profiles. As the system is highly non-linear, gradient-based algorithms are not efficient for optimization. Direct search algorithms of the MATLAB R2016b suite were explored for optimization- including the local optimizer- fminsearch and global optimizers - patternsearch, particleswarm, genetic algorithm and simulated annealing. Of these, the genetic algorithm was found to give satisfactory parameters while maintaining good convergence and was therefore used to obtain the final parameter set.

The objective function minimized is the weighted least squares.

$$\sum_{i=1}^{n}\left(\frac{\overline{[SOX]}_{m,i}-[SOX]_{p,i}}{\delta_i}\right)^2 \quad (1)$$

The first term is the mean experimental value for that particular time instant, while the next term is the model predicted value. The denominator indicates the variance, obtained from the experimental data. Additionally, bound constraints of 10% around specified values are imposed on each of the parameters to aid the optimizer in its search and to ensure that the values are biologically meaningful. These bounds signify the biological limits for each of the parameters and were obtained from literature, where available, or assumed to be within a limit for similar biological components. In cases where literature was not available, these bounds were determined so as to fit the order of the terms in the system of equations using the initial concentrations for the concentration terms.

Robustness analysis: To determine the robustness of the system, it is important to characterize the variations in behaviour of the various species, on minor variations in the kinetic parameters and initial concentrations. Further, identification of the parameters that affect the system the most could be useful as control units or targets in future experimentation, whereas the parameters that affect the system the least could be used for model reduction purposes. As we are dealing with a large system - with 8 species and 23 parameters, it is useful to define a sensitivity metric to compare the effect of each parameter on the system.

We construct a parameter sensitivity matrix S_{8x23} comprising the relative least squares sum error for each species on perturbing each parameter individually, computed for all species and parameters.

$$S(j,k) = \sum_{i=1}^{n} \left(1 - \frac{[C_j]_{P_{k1},i}}{[C_j]_{P_{k2},i}} \right)^2 \tag{2}$$

Here, i is an index for the data representing each time step, C indicates concentration of a species, j is an index over the concentrations of the 8 species present, P indicates parameter and k is an index over the 23 parameters present. n is the total number of time points in the given data set. The variables k_1 and k_2 represent the values for the parameter k before and after the required perturbation is given to the parameter.

The concentration affected the most by the parameter P_k is calculated as:

$$Cmax_{1x23} = \max S(:, k) \tag{3}$$

Each parameter was increased (and decreased) in steps of 1% until the robustness bound for $Cmax_{1x23}$ was reached. The final sensitivity metric used to compare the effect of different parameters on the system is the percentage increase and decrease required to change $Cmax$ beyond the robustness bound specified.

Tables

Supplementary Table S1. Initial concentrations for the model

**Species**	**Concentration (μM)**	**Comments /References**
MD	6	Medium concentration
SQ	1×10^{-6}	Small non negative value assigned
SOX	5.34×10^{-1}	Valued obtained from experimental data
MnSOD	4×10^{-1}	Value used in the model by the authors[1].
SOD_2	1×10^{-6}	Small non negative value assigned
p53	1.24×10^{-1}	Reported value[2]
p53-P	1×10^{-6}	Small non negative value assigned
ERK_{2c}	3	Reported value[3]
ERK_{2n}	1×10^{-6}	Small non negative value assigned
NADPH	1×10^{-1}	Value assumed within physiological limits
O_2	2×10^{1}	Reported value[4]
DNA	4.67×10^{5}	Calculated for 3 billion bp and a cell volume corresponding to a diameter of 16μm[5]

Supplementary Table S2. Optimized parameter values

Parameter	Optimized value	Units	Comments
A	2.25×10^{-1}	μM	Model defined parameter
L	1.40×10^{-2}	h^{-1}	Model defined parameter
k_2	4.66×10^{-4}	h^{-1}	Optimized for the model as literature values were not available for physiological pH and Temperature
K_{SOX}	9.83×10^{-6}	μM	
k_{dp}	2.97×10^{-3}	h^{-1}	Values calculated according to the information given in the reference[6] for mouse or sea urchin. Used in their model by authors
k_{dm}	2.79×10^{-3}	h^{-1}	
k_{tr}	1.32×10^{3}	h^{-1}	
I_m	7.26×10^{2}	h^{-1}	
k_3	3.96×10^{1}	h^{-1}	Assumed within physiological values for similar enzymes
K_{p53p}	3.05×10^{1}	μM	Studies on a similar enzyme[7]
K_{p53}	9.41×10^{-1}	μM	Studies on a similar enzyme[8].
v_{m4}	6.52×10^{5}	μM h^{-1}	Studies on a similar enzyme[7]
k_{t1}	6.34×10^{-4}	μM^{-1} h^{-1}	Value determined for first order rate constant[3]. The current model assumes second order kinetics dependent on the concentration of SOX as well
n_v	8.24×10^{-2}	-	Reported values[9]
n_a	1.84	-	Reported values[10]
K_N	3.43×10^{1}	μM	Reported values[11]
Vm1	4.63×10^{-1}	μM h^{-1}	Values determined assuming MM Kinetics[12].
K_M	1.76	μM	
k_1	8.59×10^{6}	μM^{-1} h^{-1}	Reported values[13]
k_{m1}	1.29×10^{5}	μM^{-1} h^{-1}	
v_{m5}	1.08×10^{-1}	μM h^{-1}	Assumed within physiological limits for similar enzymes
K_{NADP^+}	9.97×10^{-2}	μM	
k_p	1×10^{-6}	h^{-1}	Assumed within physiological limits

Supplementary Table S3. Optimized phase values

Case	Phase (radian)
MD=0	-0.18π
MD=3	0.86π
MD=6	-0.18π
MD=9	-1.19π
MD=12	-0.37π
MD=15	-0.62π
Without p53<br>(All MD conc.)	1.5π

Supplementary Table S4. Parameter robustness limits

Parameter	Lower limit	Upper limit	% decrease	% increase	Species affected
A	9.66×10^{-2}	3.55×10^{-1}	57	58	SOX
L	4.2×10^{-4}	4.32×10^{-2}	97	209	SOX
k_2	4.66×10^{-6}	3.03×10^{-3}	99	549	SOX
K_{sox}	9.82×10^{-9}	1.08×10^{-5}	99	999	SOX
k_{tr}	4.49×10^{2}	2.190×10^{3}	66	66	MnSOD
k_{dp}	2.97×10^{-5}	1.55×10^{-2}	99	422	MnSOD
Im	5.30×10^{2}	9.22×10^{2}	27	27	SOD2
k_{dm}	2.79×10^{-5}	2.99×10^{-2}	99	973	SOD2
k_3	3.33×10^{1}	4.51×10^{1}	16	14	SOD2
K_{p53p}	3.05×10^{-1}	3.35×10^{2}	99	999	SOX
K_{p53}	8.09×10^{-1}	1.14	14	21	SOD2
v_{m4}	5.74×10^{5}	7.76×10^{5}	12	19	SOD2
K_{t1}	5.32×10^{-4}	7.23×10^{-4}	16	14	SOD2
n_v	8.16×10^{-2}	8.32×10^{-2}	1	1	SOD2
n_a	1.82	1.86	1	1	SOD2
Phi	-1.64	-2.22	15	15	SOX
v_{m1}	4.63×10^{-3}	1.3	99	181	SOX
k_{na}	1.2×10^{1}	3.76×10^{2}	65	999	SOX
k_m	1.76×10^{-2}	1.93×10^{1}	99	999	SOX
k_1	7.13×10^{6}	1.08×10^{7}	17	26	SQ
k_{m1}	1.02×10^{5}	1.56×10^{5}	21	21	SQ
v_{m5}	2.16×10^{-3}	1.19	98	999	NADPH
K_{NADP^+}	9.97×10^{-4}	1.1	99	999	NADPH

Figures

$$[ERK]_n + n_v[ERK]_c = [ERK]_{total}$$

$$[NADPH] + [NADP^+] = [NADP]_{total}$$

$$[O_2] = [O_2]_{total}$$

$$[DNA] = D_n$$

Supplementary Figure S1: Conservation relations used in the model. The square brackets indicate the concentration of the respective species. The subscripts n and c for ERK denote the nuclear and cytoplasmic concentrations of ERK.

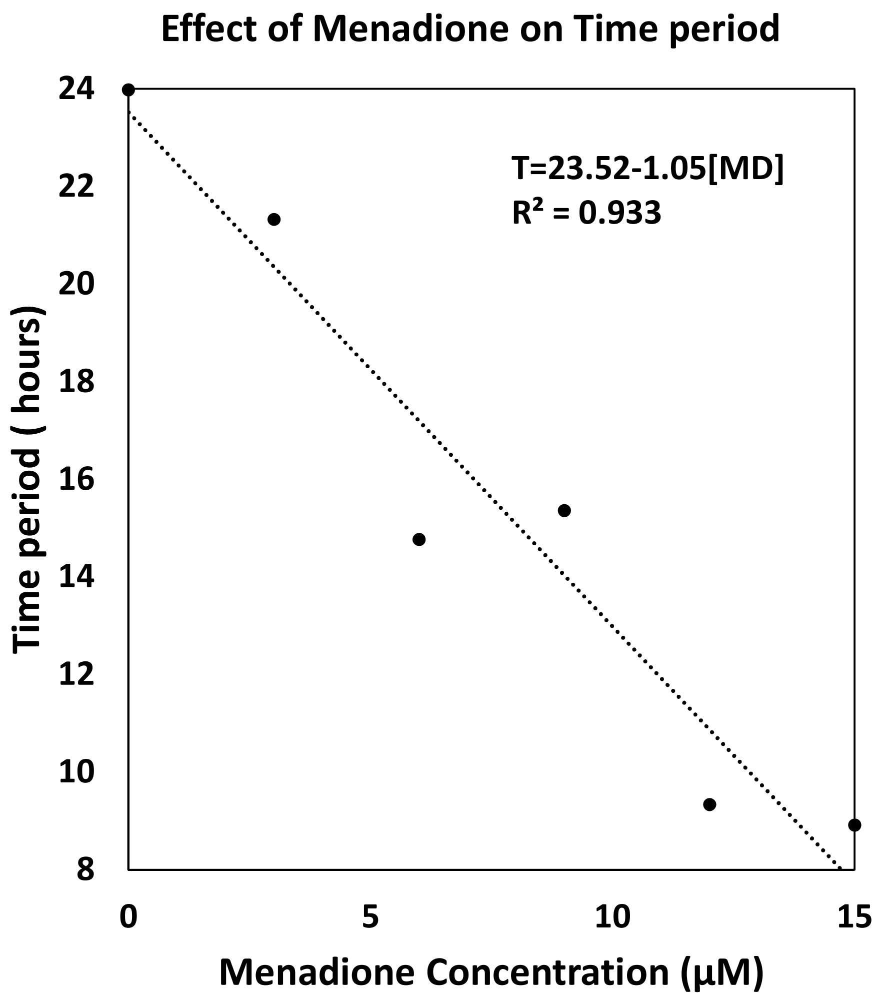

Supplementary Figure S2: Linear regression of time period of SOX oscillations in presence of p53 over MD concentration in HCT116 wt.

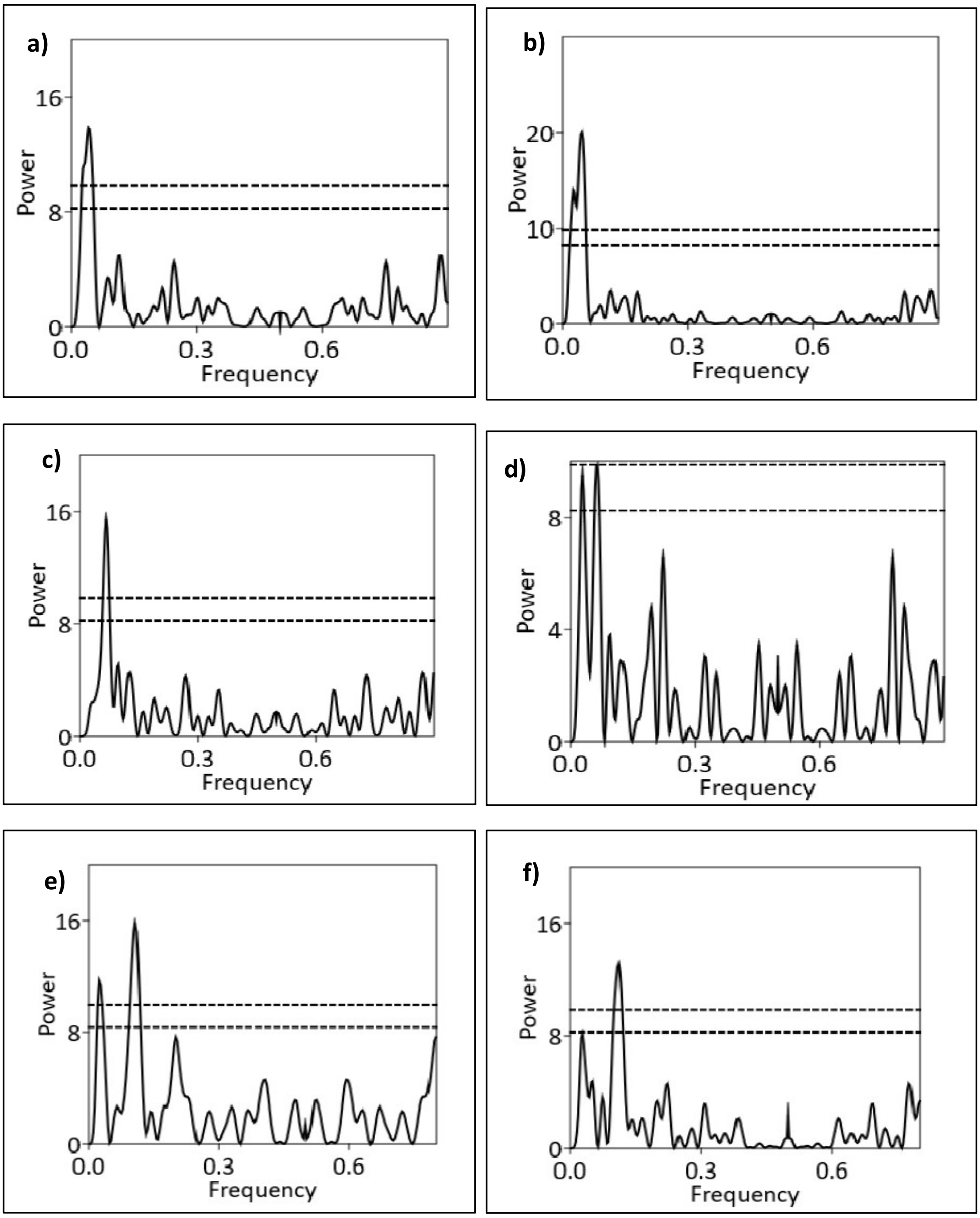

Supplementary Figure S3: si-SOX time series power spectral graphs of frequencies generated in the Lomb–Scargle Periodogram for HCT116 wt cells a) untreated control ($p=1.8\times10^{-4}$) b) 3μM menadione ($p=3.9\times10^{-7}$) c) 6μM menadione ($p=3.7\times10^{-5}$) d) 9μM menadione ($p=1\times10^{-2}$) e) 12μM menadione ($p=3\times10^{-}$

[5]) f) 15µM menadione ($p=3.9\times10^{-4}$). The bottom and top dotted lines represent $p<0.05$ and $p< 0.01$ respectively.

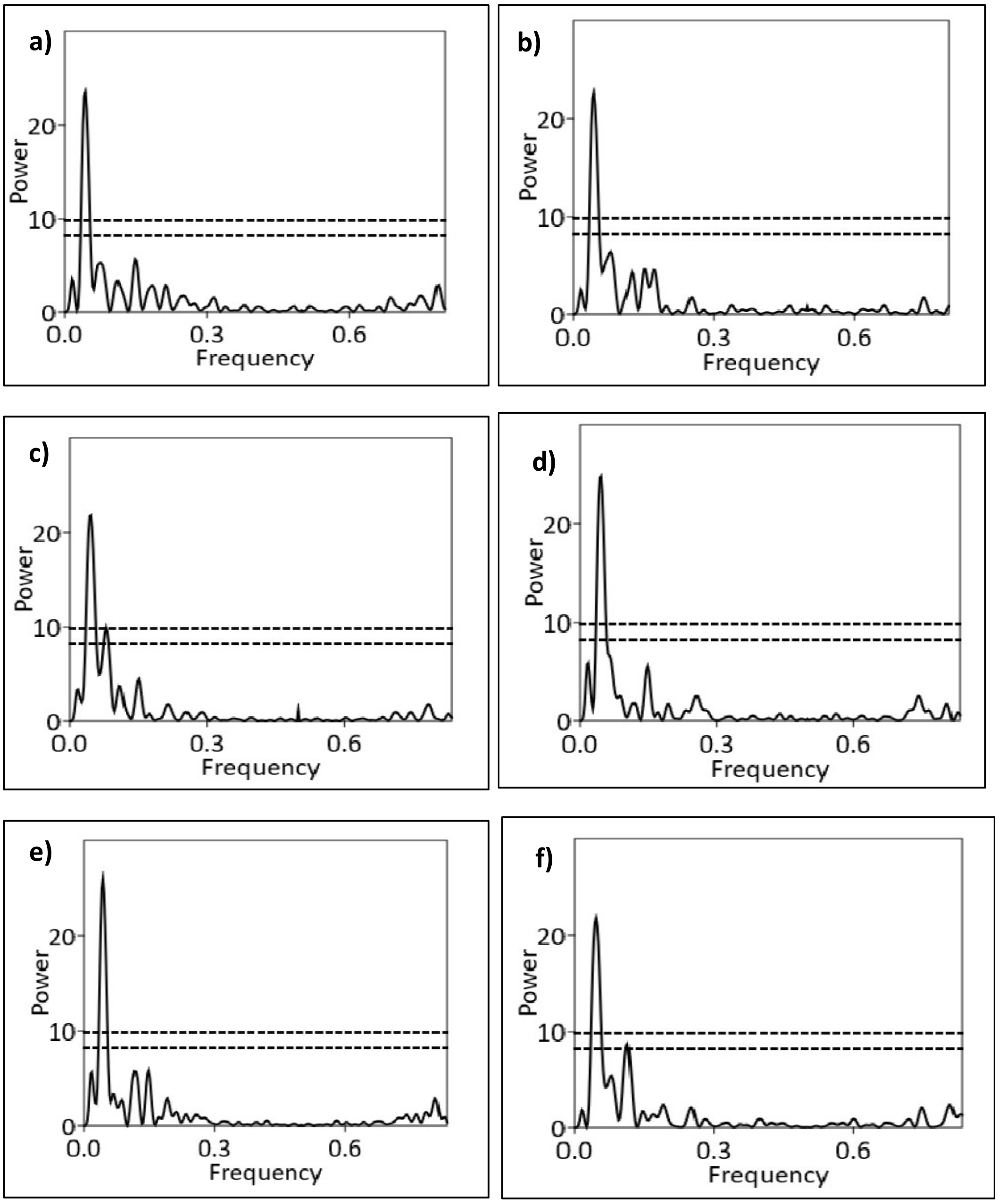

Supplementary Figure S4: si-SOX time series power spectral graphs of frequencies generated in the Lomb–Scargle Periodogram for HCT116 p53-/- cells a) untreated control ($p=1.2\times10^{-8}$) b) 3µM menadione ($p=2.9\times10^{-8}$) c) 9µM menadione ($p=6.2\times10^{-8}$) d) 12µM menadione ($p=3.3\times10^{-9}$) e) 15µM menadione ($p=1\times10^{-9}$) f) 30µM menadione ($p=6.8\times10^{-8}$). The bottom and top dotted lines represent $p<0.05$ and $p< 0.01$ respectively.

a)

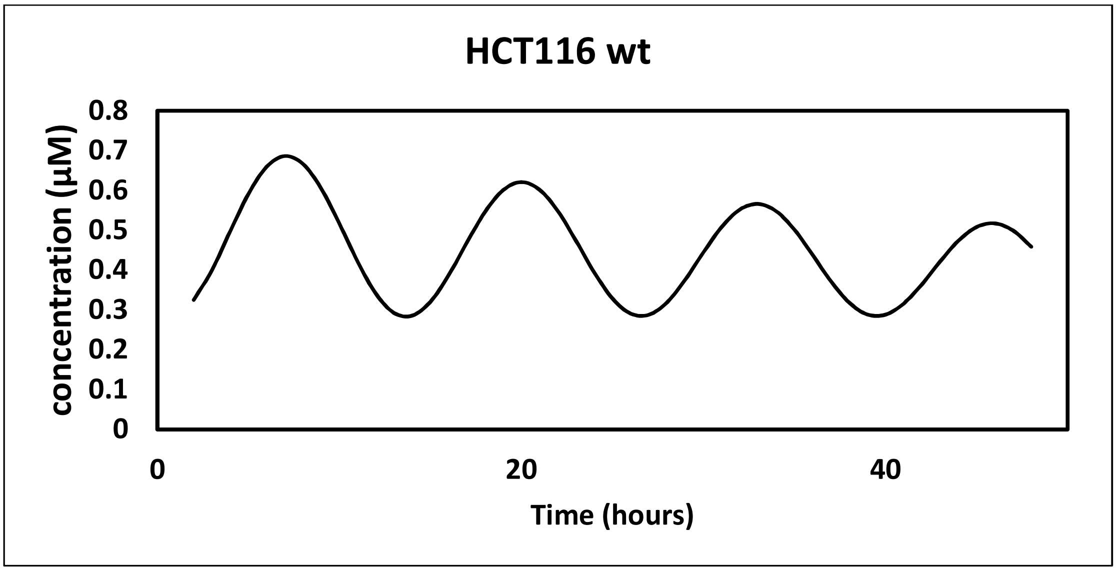

b)

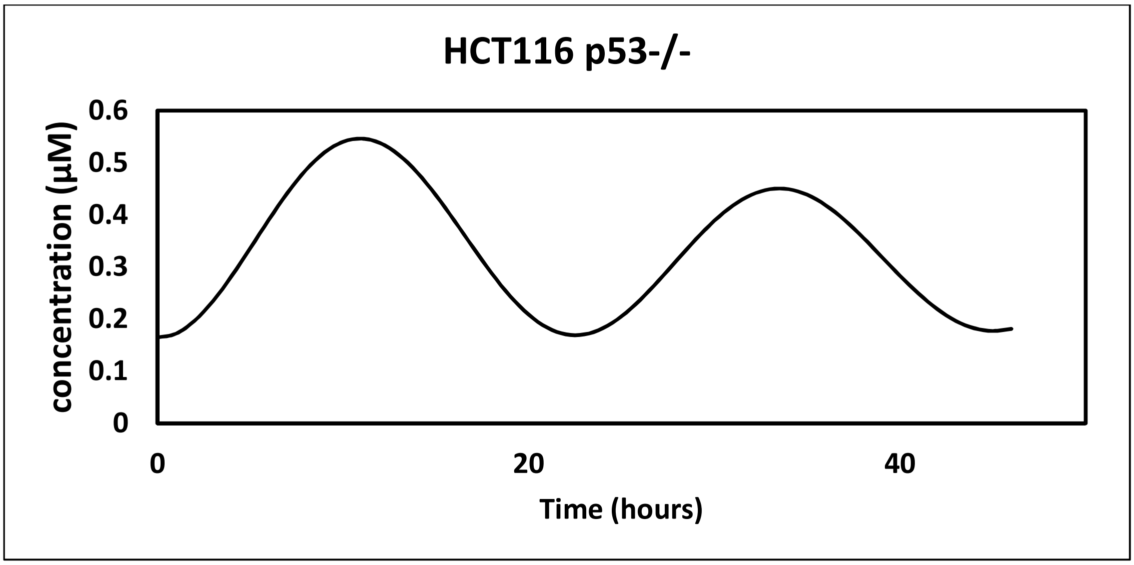

Supplementary Figure S5. Model predictions for new MD cases.

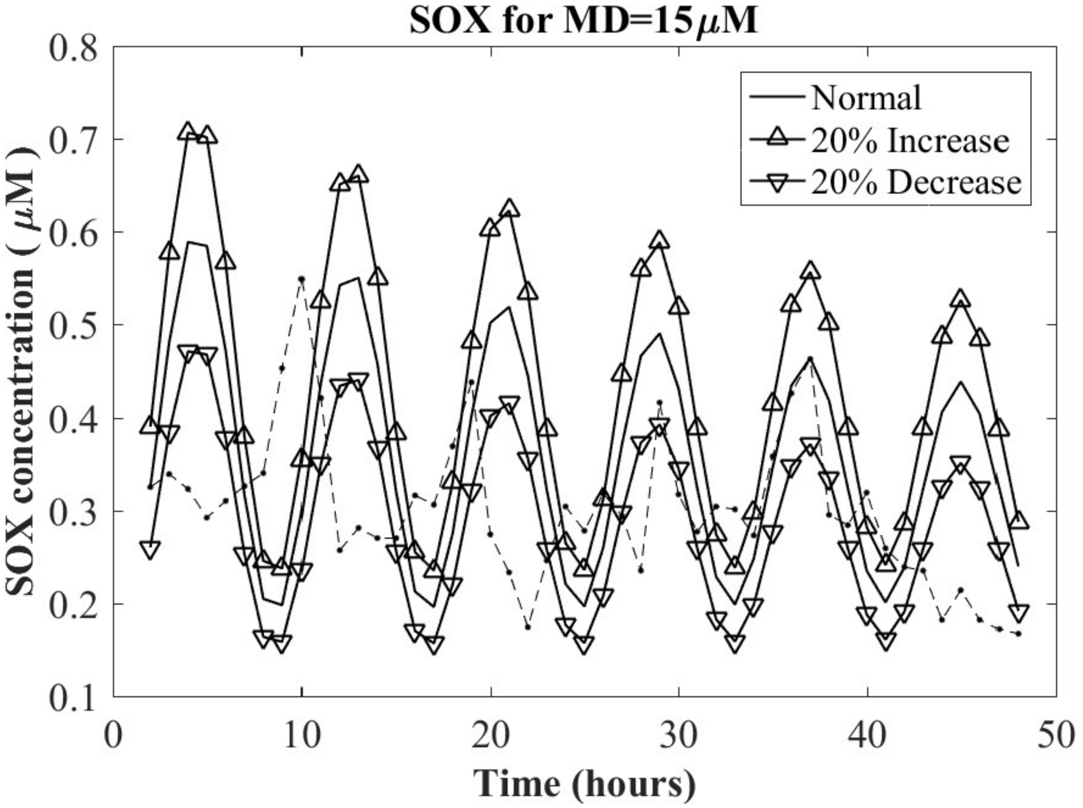

Supplementary Figure S6. Robustness bounds. The (-•-) line represents experimental data.

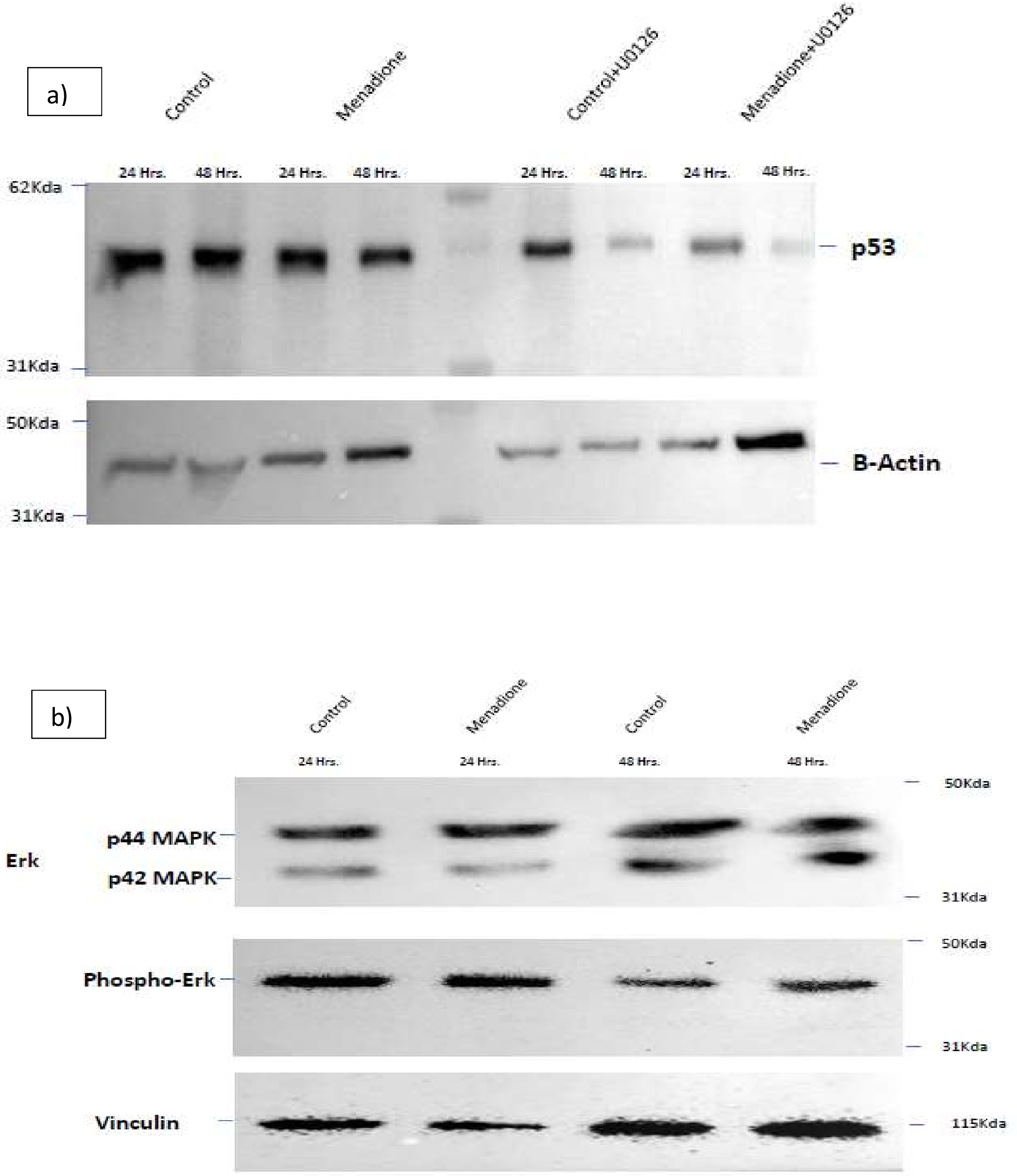

Supplementary Figure S7: Western blot images for a) p53 (top panel) and b) ERK 1/2 and Phospho- ERK. The full blots are provided in Supplementary Fig S14.

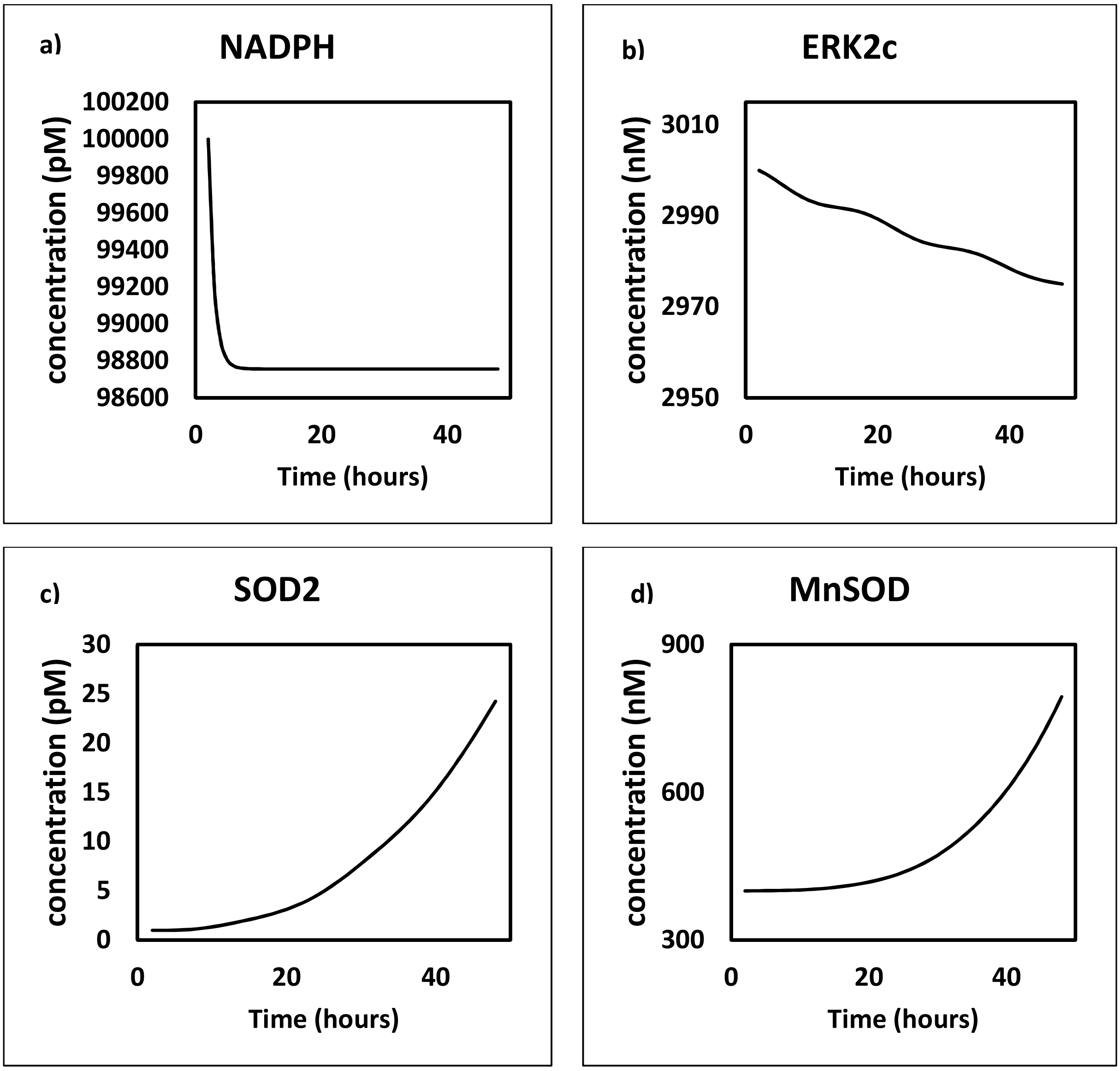

Supplementary Figure S8. Species Behaviour for MD = 6 µM with p53

a)

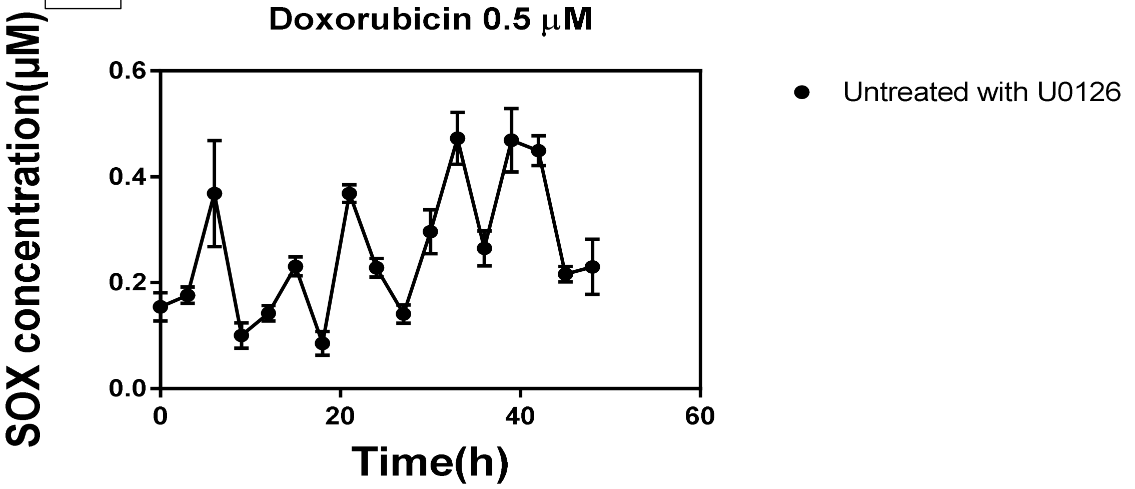

b)

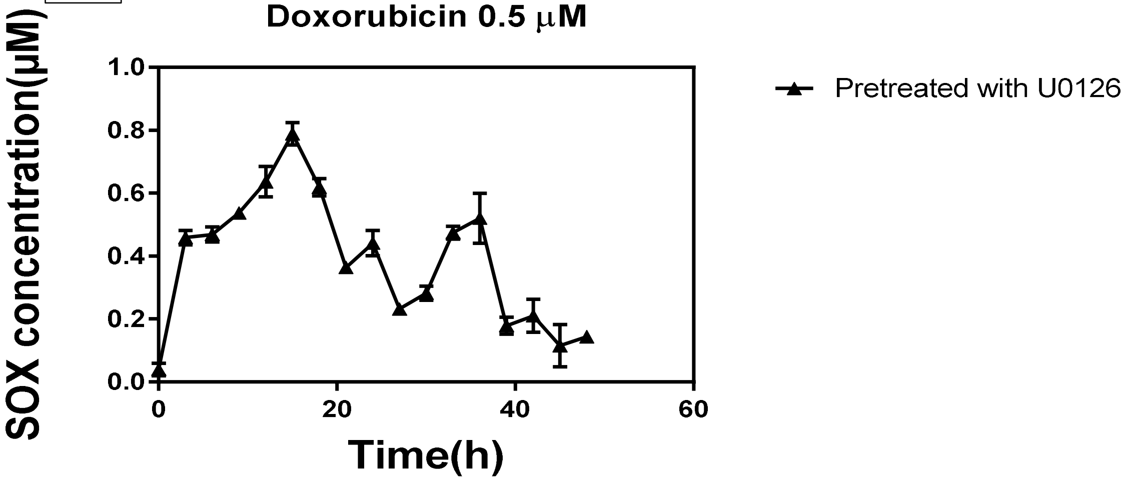

Supplementary Figure S9. Temporal variation of SOX values for HCT116 wt cells treated with doxorubicin, 0.5 μM for a) untreated with U0126 b) pretreated for 2h with U0126. Doxorubicin induces a SOX rhythm reset to 8.9 h from the near circadian rhythm of untreated control. The inhibition of ERK activation by U0126 removes this doxorubicin induced reset and gives a SOX rhythm of 22.7h.

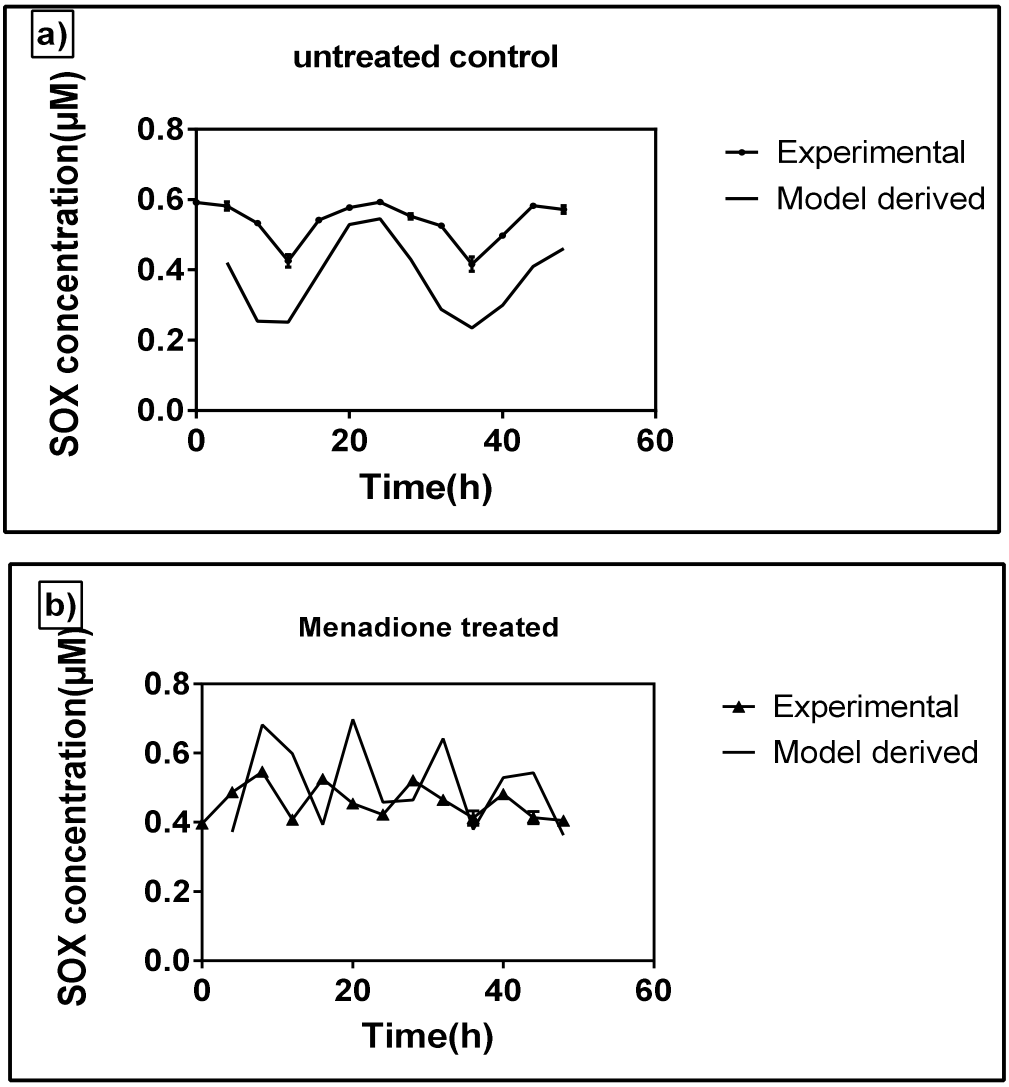

Supplementary Figure S10. Comparison of experimental and model derived SOX values for HepG2 for a) untreated control b) cells treated with menadione at IC_{50} concentration. The parameters a and b were tuned slightly to obtain the model derived rhythm. HepG2 shows an inherent SOX rhythm of 25.64h reset to 11h after menadione treatment.

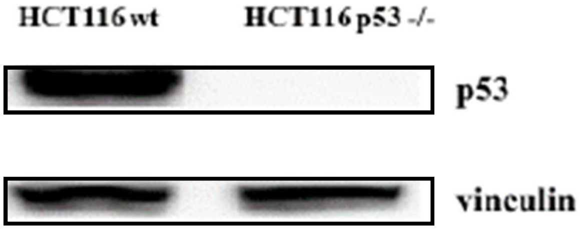

Supplementary Figure S11: validation of the p53 status of the cell lines by western blot. Original uncropped gel images are given in Supplementary Fig S11.

HCT116 p53-/-

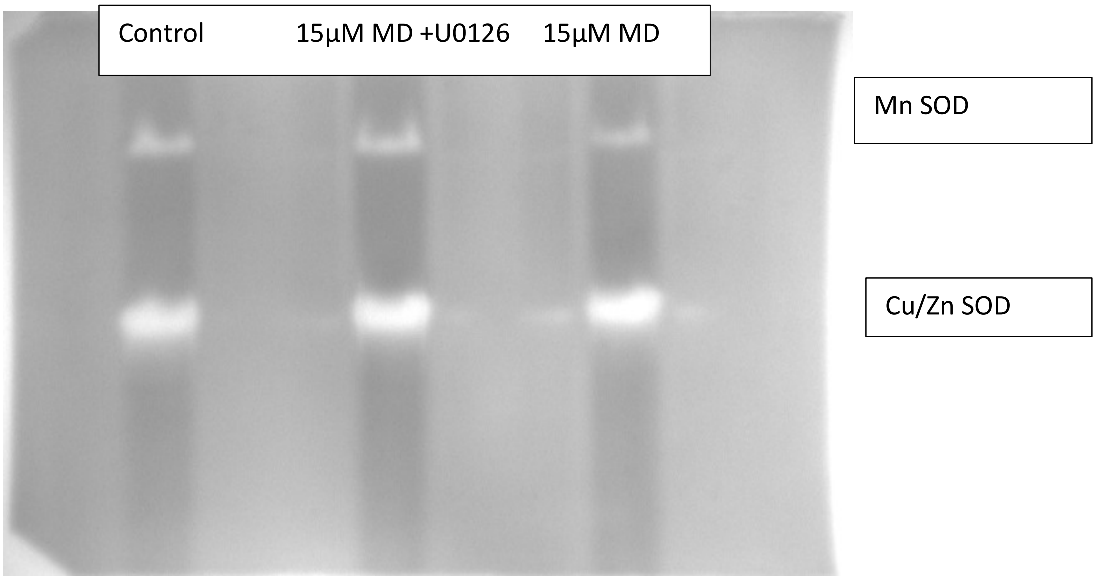

HCT116 wt

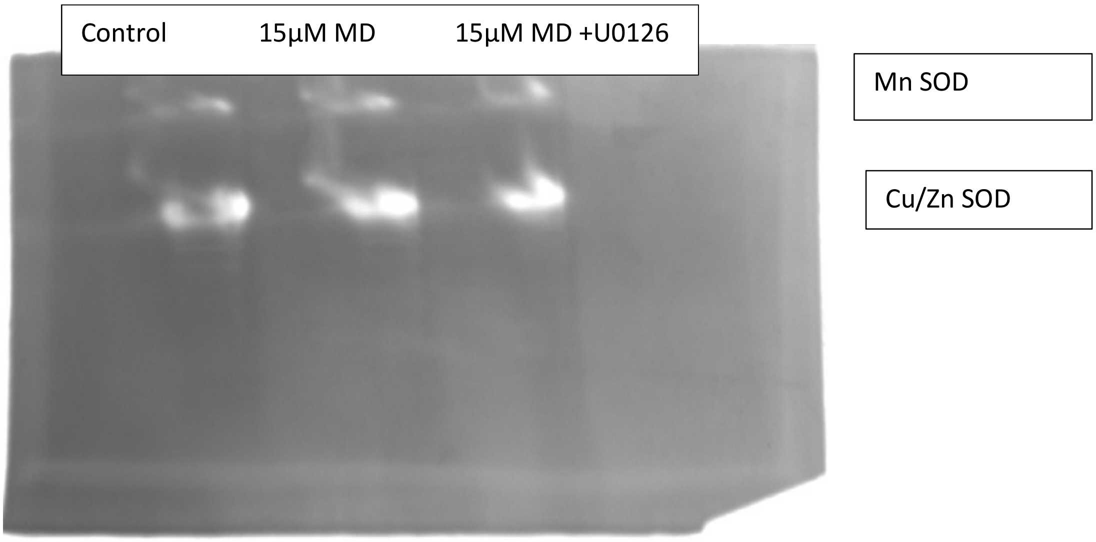

Supplementary Figure S12: Original gel image for MnSOD native gel assay. The top lane shows the MnSOD levels and the bottom lane shows the Cu/Zn SOD levels. The images were captured using GelDoc and analyzed using ImageJ software.

a)

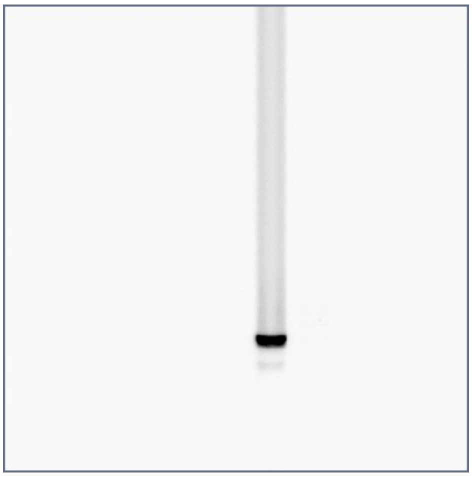

b)

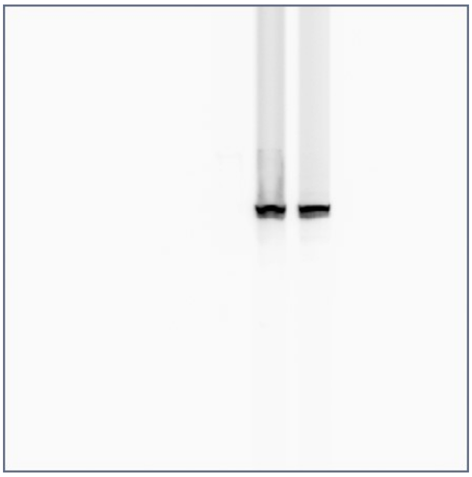

Supplementary Figure S13: Original gel images (uncropped) for a) p53 b) vinculin for validation of p53 status of the cell lines by western blot.

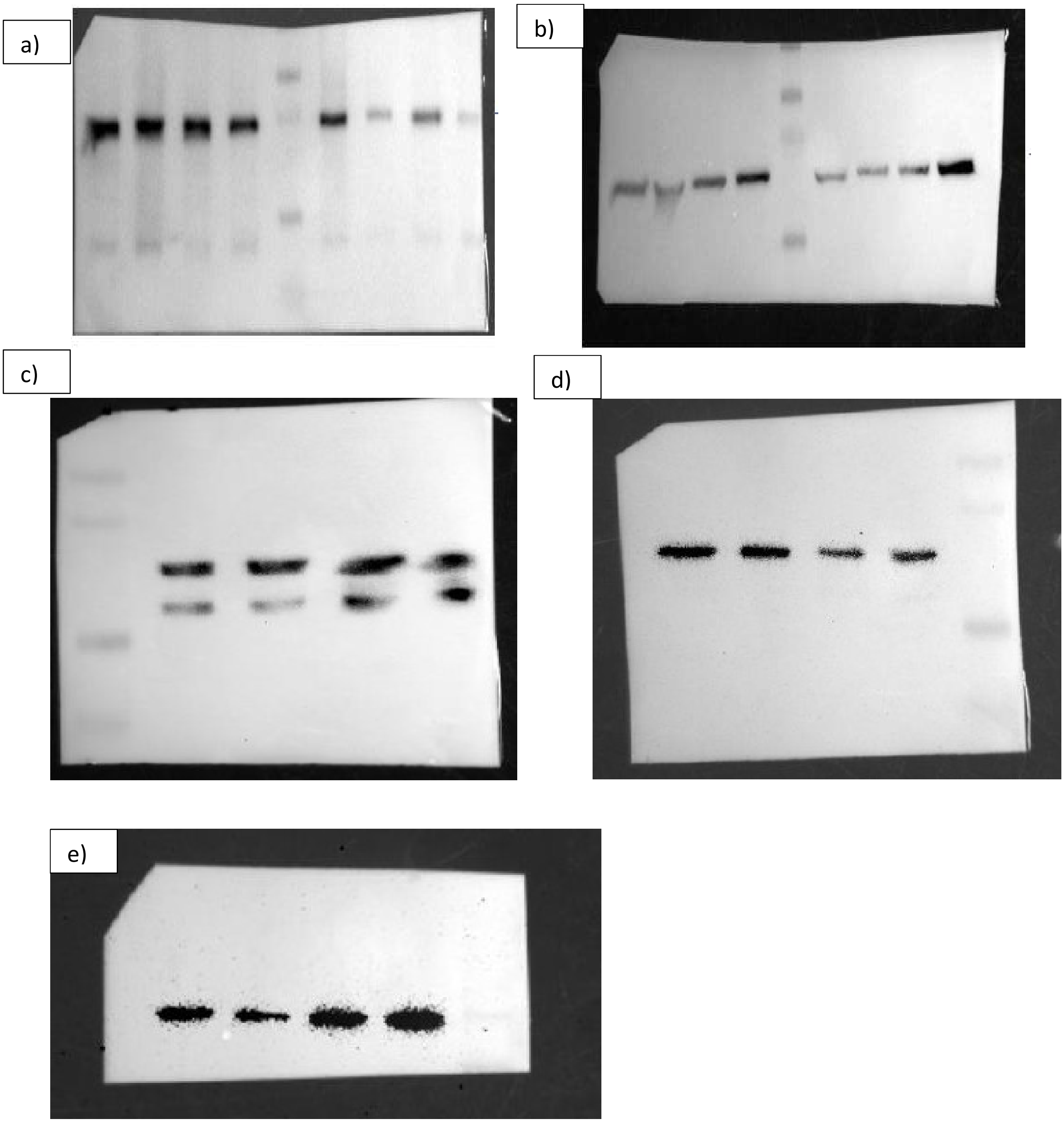

Supplementary Figure S14: Full blot images for a) p53 b) β-Actin c) ERK d) Phospho ERK and e) Vinculin.

Supplementary References

1. Hsu, J. L.et al. Catalytic properties of human manganese superoxide dismutase. *J Biol Chem.* **271**, 17687-17696 (1996)
2. Ma, L. *et al.* A plausible model for the digital response of p53 to DNA damage. *Proc. Natl. Acad. Sci. U. S. A.* **102,** 14266–71 (2005).
3. Fujioka, A. *et al.* Dynamics of the Ras/ERK MAPK cascade as monitored by fluorescent probes. *J. Biol. Chem.* **281,** 8917–8926 (2006).
4. Wagner, B. A., Venkataraman, S. & Buettner, G. R. *The Rate of Oxygen Utilization by Cells*. *Free Radic Biol Med* **51,** (2011).
5. Tahara, M. *et al.* The Use of Olaparib (AZD2281) Potentiates SN-38 Cytotoxicity in Colon Cancer Cells by Indirect Inhibition of Rad51-Mediated Repair of DNA Double-Strand Breaks. *Mol. Cancer Ther.* **13,** 1170–1180 (2014).
6. Ben-Tabou de-Leon, S. Perturbation analysis analyzed-mathematical modeling of intact and perturbed gene regulatory circuits for animal development. Dev Biol. **344**, 1110-1126 (2010).
7. Goloudina, a. R. *et al.* PNAS Plus: Wip1 promotes RUNX2-dependent apoptosis in p53-negative tumors and protects normal tissues during treatment with anticancer agents. *Proc. Natl. Acad. Sci.* **109,** E68–E75 (2012).
8. Barcia, R. *et al.* Kinetic properties of p53 phosphorylation by the human vaccinia-related kinase 1. *Arch. Biochem. Biophys.* **399,** 1–5 (2002).
9. Huber, M. D. & Gerace, L. The size-wise nucleus: Nuclear volume control in eukaryotes. *J. Cell Biol.* **179,** 583–584 (2007).

10. Follis, A. V. *et al.* The DNA-Binding Domain Mediates both Nuclear and Cytosolic Functions of p53. *Nat Struct Mol Biol* **21,** 535–543 (2014).
11. Simtchouk, S., Eng, J. L., Meints, C. E., Makins, C. & Wolthers, K. R. Kinetic analysis of cytochrome P450 reductase from Artemisia annua reveals accelerated rates of NADH-dependent flavin reduction. *FEBS J.* **280,** 6627–6642 (2013).
12. Bayol-Denizot, C., Daval, J. L., Netter, P. & Minn, A. Xenobiotic-mediated production of superoxide by primary cultures of rat cerebral endothelial cells, astrocytes, and neurones. *Biochim. Biophys. Acta - Mol. Cell Res.* **1497,** 115–126 (2000).
13. Song, Y. & Buettner, G. R. Thermodynamic and kinetic considerations for the reaction of semiquinone radicals to form superoxide and hydrogen peroxide. *Free Radic. Biol. Med.* **49,** 919–962 (2010).
